# Lingual thyroid causing dysphonia: evaluation and management. Case report

**DOI:** 10.1590/S1516-31802004000200007

**Published:** 2004-03-01

**Authors:** Alfio José Tincani, Antonio Santos Martins, André Del Negro, Priscila Pereira Costa Araújo, Gilson Barretto

**Keywords:** Thyroid, Dysphonia, Head and neck tumors, Malformations, X-Ray Computed Tomography, Tireóide, Disfonia, Tomografia computadorizada por raio x

## Abstract

**CONTEXT:**

Lingual thyroid gland is a rare clinical entity that is caused by the failure of the thyroid gland to descend to a normal cervical location during embryogenesis. The occurrence of an ectopic thyroid gland located at the base of the tongue may cause problems for the patient, with symptoms of dysphagia, dysphonia, upper airway obstruction or even hemorrhage at any time from infancy through adulthood.

**CASE REPORT:**

We report on a case of lingual thyroid gland in a 41-year-old female patient. The embryology and diagnosis of ectopic thyroid are discussed and its management is outlined. Features of the diagnostic and therapeutic evaluation are described with attention to the clinical findings, laboratory tests, thyroid scan and computed tomography imaging studies employed in the confirmation of diagnosis and planning of appropriate treatment. The history of the condition is reviewed and a treatment strategy is outlined. Surgical excision of the gland is reserved for cases of gland enlargement that result in compromised airways (dysphagia or dysphonia) or recurrent hemorrhage.

## RESUMO

**CONTEXTO:**

Tireóide lingual é uma entidade rara, devida a falha na migração da glândula em sua fase embriogênica. A presença da glândula tireóide na base da língua pode acarretar sérios problemas aos pacientes, como disfagia e disfonia, obstrução de vias aéreas superiores e até hemorragia, em qualquer fase da vida.

**RELATO DE CASO:**

Apresentamos um caso de tireóide lingual em paciente de 41 anos de idade, discutindo-se a embriogênese, diagnóstico e conduta adequada para tratamento. Elementos para o diagnóstico e avaliação terapêutica são descritos com especial atenção aos achados clínicos, testes laboratoriais, além de metodologia de imagem como medicina nuclear e tomografia computadorizada, realizados para confirmação diagnóstica e planejamento da melhor conduta operatória. A excisão cirúrgica da tireóide ectópica é reservada para casos de aumento glandular, que podem resultar em disfunção das vias aéreas superiores (disfonia ou disfagia), além de hemorragia recorrente.

## INTRODUCTION

A lingual thyroid gland is an ectopic thyroid tissue located on the midline of the base of the tongue.^1-4^ When not located in the second, third and fourth tracheal rings along the midline of the ventral portion of the neck, the thyroid gland is characterized as ectopic.^2-4^

Although the pathogenesis of lingual thyroid is unclear, some authors have postulated that maternal antithyroid immunoglobulins may impair gland de- scent.^2,3^ Ectopic thyroid glands may be found within four general groups in the upper aerodigestive tract. These categories are based upon the natural descent of the thyroid from its embryological starting point at the base of the tongue to its final resting position, anterior to the trachea: lingual (most frequent), sublingual, thyroglossal and intralaryngotracheal.^2,3^ Lingual thyroid is found in approximately 1 in 100,000 people and there is a marked female sex predominance ratio of 4:1 to 7:1.^2,3^ The location of the ectopic thyroid can be identified by thyroid scan. Other rare locations described in the literature are the oropharynx, esophagus, pericardium, mediastinum and diaphragm.^2,3^ Only rarely there is a report of carcinoma originating in a lingual thyroid gland.^2^

## CASE REPORT

A 41-year-old woman was referred with a history of dysphonia and mild dysphagia to solid foods. She also reported that she had been using thyroid hormone replacement for eight years, as prescribed by a doctor, due to thyroid dysfunction. She was unaware of her thyroid status.

Upon physical examination, it was noticed that she had a 6 × 5 cm smooth, rubbery and reddish mass on the midline at the base of the tongue, covered by normal mucosae, just posterior to the *circumvallate papillae*. During neck examination, the thyroid gland was not palpable.

**Figure 1:**
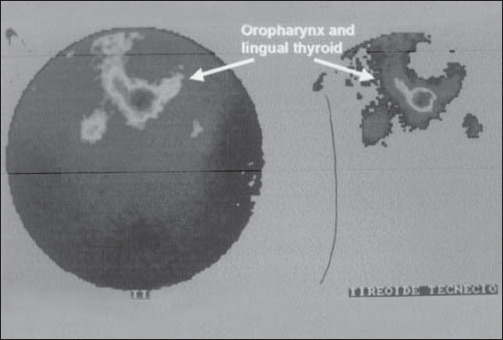
Thyroid scan with technetium (Tc99m) revealing isotope uptake at the base of the tongue and no uptake in the normal thyroid location (uptake in 2 hours: 5.8%; uptake in 6 hours: 6.3%).

Thyroid function tests demonstrated normal T3, T4 and thyroid-stimulating hormone levels. Other laboratory tests were within normal limits. Additional testing included technetium (Tc99m) thyroid scan, which revealed isotope uptake at the base of the tongue and no uptake in the normal thyroid location (Figure 1).

**Figure 2:**
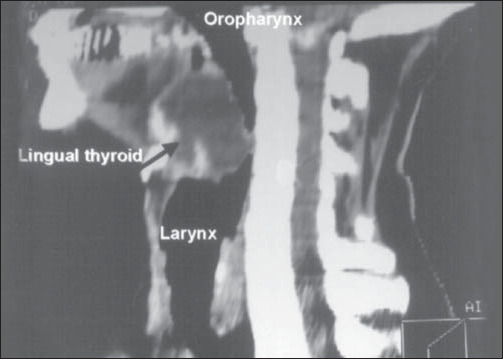
Computed tomography imaging scan demonstrating an oval-shaped mass at the base of the tongue causing sub-occlusion in the oropharynx.

A computed tomography imaging scan revealed an oval-shaped mass at the base of the tongue causing sub-occlusion in the oropharynx and absence of the normal thyroid gland in its usual location (Figure 2). No other imaging tests, like magnetic resonance imaging (MRI) or ultrasound, were performed.

The patient was diagnosed as having lingual thyroid and was submitted to surgical resection of the gland under general anesthesia. Intubation was performed, with fiberoptic endoscopy and insertion of a nasotracheal tube.

**Figure 3:**
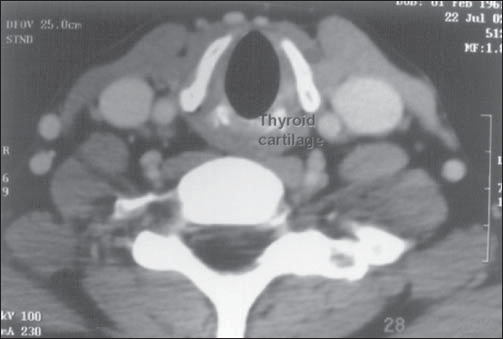
Computed tomography of the larynx, demostrating thyroid absence.

The ectopic thyroid was resected by means of an oral approach (Figure 3), using a harmonic scalpel to diminish the bleeding. No temporary tracheostomy was needed. Her postoperative evolution was uneventful, and voice and diet restoration were immediate.

## DISCUSSION

Lingual thyroid gland is a rare clinical entity caused by the failure of the gland to descend from its anlage, early in the course of embryogenesis.^1-3^

The symptoms may be varied, most of them related to oropharyngeal obstruction, and may include dysphagia (mild or severe), dyspnea and, as reported by us, dysphonia.^4^ Stridor is most common in neonates.^2,3^ A rarely described symptom is bleeding.^3^ Depending on the patient's age, the symptoms may be drastic: infants and young children whose lingual thyroid is detected via routine screening may suffer from failure to thrive and mental retardation, or even severe respiratory distress, resulting in a medical emer- gency.^3^ Other cases may present with onset of slowly progressing dysphagia and symptoms of oropharyngeal obstruction before or during puberty, or even during pregnancy. This occurs as a response to the increased demand for thyroid hormone in these hypermetabolic states.

The evaluation of such patients includes thorough head and neck examination with special attention to the base of the tongue. Endoscopic examination of the upper airway is opportune, in order to determine gland size and whether ulceration or hemorrhage is present, and to view the larynx and hypopharynx. Palpation of the neck is absolutely essential, in order to check the presence or absence of the thyroid gland in its normal position. Thyroid function tests must also be performed, but these often demonstrate normal gland functions.^1-4^

Technetium scanning confirms the presence of ectopic thyroid tissue at the base of the tongue. In our case report, the computed tomography (CT) scan demonstrated the size of the gland at the base of the tongue, its infiltration of the muscle and the absence of normal thyroid tissue in the neck (Figure 3). In this situation and in our opinion, an ultrasound examination is unnecessary.

Although controversial, in small oligosym- ptomatic lingual thyroid glands, clinical treatment may be attempted, using suppressive therapy with exogenous thyroid hormone.^1,2^

**Figure 4:**
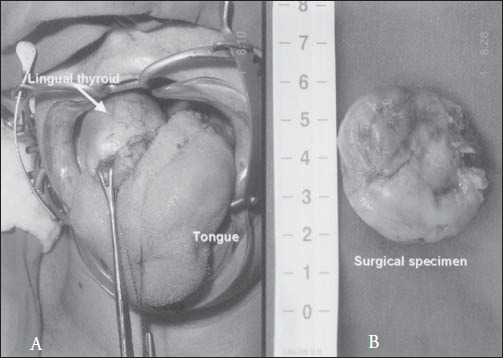
Lingual thyroid in the base of the tongue ready for resection via oral approach (A). The surgical specimen after resection (B).

The surgical management of lingual thyroid depends on the severity of the symptoms present. Preoperative tracheostomy and nasotracheal intubation are both effective. In our case, patient intubation was done by means of fiberoptic endoscopy with nasotracheal tube access, thus not requiring any temporary tracheostomy (Figure 4).

Suprahyoid access, or combined cervical and intraoral access,^1,2,4^ is described as excellent for a surgical approach. We achieved good exposure by transoral route without intra or postoperative complications, and this was very important for immediate patient recovery, with total excision of the ectopic thyroid.

## CONCLUSION

Lingual thyroid is a rare entity that may cause serious problems for the patient. When diagnosed in adults, thyroid function tests and radionuclide scanning are essential. Computed tomography scans are necessary for planning surgical intervention and the approach.

Although other types of surgical access have been described,^1-4^ the transoral approach, in our opinion, provides good exposure and is less traumatic for the patient, with better postoperative recovery.
